# Diverse genome structures of *Salmonella paratyphi *C

**DOI:** 10.1186/1471-2164-8-290

**Published:** 2007-08-27

**Authors:** Wei-Qiao Liu, Gui-Rong Liu, Jun-Qian Li, Guo-Min Xu, Danni Qi, Xiao-Yan He, Juan Deng, Feng-Min Zhang, Randal N Johnston, Shu-Lin Liu

**Affiliations:** 1 Microbiology, Peking University Health Science Center, Beijing, China; 2 Microbiology and Infectious Diseases, University of Calgary, Calgary, Canada; 3 Microbiology, Harbin Medical University, Harbin, China; 4 Biochemistry and Molecular Biology, University of Calgary, Calgary, Canada

## Abstract

**Background:**

*Salmonella paratyphi *C, like *S. typhi*, is adapted to humans and causes typhoid fever. Previously we reported different genome structures between two strains of *S. paratyphi *C, which suggests that *S. paratyphi *C might have a plastic genome (large DNA segments being organized in different orders or orientations on the genome). As many but not all host-adapted *Salmonella *pathogens have large genomic insertions as well as the supposedly resultant genomic rearrangements, bacterial genome plasticity presents an extraordinary evolutionary phenomenon. Events contributing to genomic plasticity, especially large insertions, may be associated with the formation of particular *Salmonella *pathogens.

**Results:**

We constructed a high resolution genome map in *S. paratyphi *C strain RKS4594 and located four insertions totaling 176 kb (including the 90 kb SPI7) and seven deletions totaling 165 kb relative to *S. typhimurium *LT2. Two rearrangements were revealed, including an inversion of 1602 kb covering the *ter *region and the translocation of the 43 kb I-CeuI F fragment. The 23 wild type strains analyzed in this study exhibited diverse genome structures, mostly as a result of recombination between *rrn *genes. In at least two cases, the rearrangements involved recombination between genomic sites other than the *rrn *genes, possibly homologous genes in prophages. Two strains had a 20 kb deletion between *rrlA *and *rrlB*, which is a highly conservative region and no deletion has been reported in this region in any other *Salmonella *lineages.

**Conclusion:**

*S. paratyphi *C has diverse genome structures among different isolates, possibly as a result of large genomic insertions, e.g., SPI7. Although the *Salmonella *typhoid agents may not be more closely related among them than each of them to other *Salmonella *lineages, they may have evolved in similar ways, i.e., acquiring typhoid-associated genes followed by genome structure rearrangements. Comparison of multiple *Salmonella *typhoid agents at both single sequenced genome and population levels will facilitate the studies on the evolutionary process of typhoid pathogenesis, especially the identification of typhoid-associated genes.

## Background

Of the over 2500 *Salmonella *serotypes recognized to date [1], about 1400 infect humans and other warm-blooded vertebrates, which are classified into *Salmonella *subgroup I (now often referred to as *Salmonella enterica *subspecies *enterica *[2-4]). Although most of these *Salmonella *serotypes cause self-limiting gastroenteritis in humans, four cause typhoid fever, a serious and potentially fatal systemic infection, including *S. typhi *(see a recent review [5]) and *S. paratyphi *A, B and C. It is a long unanswered question whether different *Salmonella *typhoid agents cause the disease by similar or distinct mechanisms. As all *Salmonella *share high levels of genetic similarity, it is possible to reveal genetic differences or similarities in pathogenicity among the *Salmonella *typhoid agents by focusing on genomic features common to some or all of the *Salmonella *typhoid agents.

The extraordinary levels of genetic similarity among salmonellae were recognized first by genomic DNA re-association experiments [6], later by physical mapping [7,8], and recently by whole genome sequencing [9-13]. In the mid-1990s, physical mapping revealed an overall common genome structure among all *Salmonella*; it also revealed special features of individual *Salmonella *lineages, such as the major genomic insertions and rearrangements of *S. typhi *[14-16] and *S. paratyphi *A [17], which were later all confirmed by whole genome sequencing [9,11,12]. Sequence comparison of the *Salmonella *genomes further reveals that about 13% of the genes in *S. typhi *are not found in *S. typhimurium *[9,10], and many of these serovar-specific genes were apparently acquired by lateral transfer of large DNA segments from other sources [18], which, if 12 kb or larger, can be resolved on the physical maps [14,17,19-22].

Several lines of evidence suggest that the typhoid agents have not evolved by vertical descent of one from another, as genetic distances are not closer among the typhoid agents than each of them to the non-typhoidal salmonellae [12,23-25]. However, they may have common or similar routes of acquisition of genes coding for the typhoid pathogenicity. Comparison of multiple typhoid agents may lead to identification of these genes.

*S. paratyphi *C is a member of serogroup C1 [2], causing typhoid in humans but, unlike *S. typhi*, also occasionally infecting animals [26]. Previously we reported I-CeuI maps on two *S. paratyphi *C strains, RKS4587 and RKS4594, which are very different in size and organization of the seven I-CeuI fragments. In several ways, *S. paratyphi *C is similar to *S. typhi*, including the possession of a large pathogenicity island, SPI7, with genes coding for the Vi (virulence) antigen and other genes potentially associated with virulence [9,14,15,27-29]. It is thus of great significance to reveal the genomic features of *S. paratyphi *C at higher resolution and involving a broader range of wild type strains, and then compare them with those of *S. typhi *to explore the evolution of the typhoid-associated pathogenicity. In this paper, we first report a high resolution genome map of a representative *S. paratyphi *C strain, RKS4594, featured with several insertions, deletions and rearrangements. We then present genome maps of additional wild type strains of *S. paratyphi *C constructed with I-CeuI, which reveal diverse genome structures among the wild type strains of *S. paratyphi *C. Some strains have deletions in the highly conserved *metE-argE *region, which have not previously been observed in any other *Salmonella *strains.

## Results

### Cleavage of *S. paratyphi *C RKS4594 genomic DNA with I-CeuI

**Figure 1:**
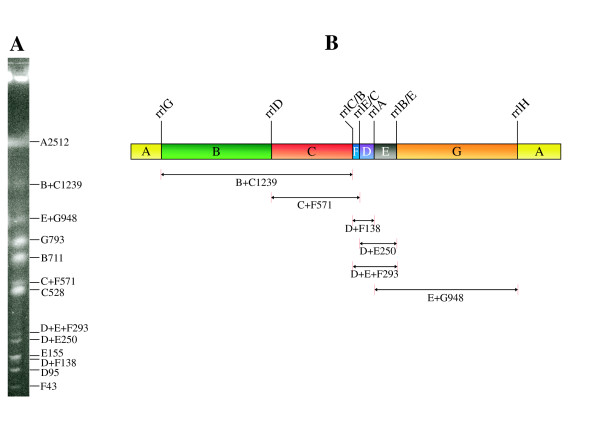
**I-CeuI mapping of *S. paratyphi *C RKS4594**. (A) PFGE separation of I-CeuI cleaved genomic DNA. See more detailed description in the text about complete and partial cleavages of the genomic DNA with I-CeuI. (B) I-CeuI cleavage map of the RKS4594 genome based on (A). The genome is circular; here it is shown as a linear map for the convenience of illustrating the mapping procedure. Only parts of fragment A (2512 kb) are shown at the ends of the linear map. The remaining 6 fragments are drawn to scale.

I-CeuI is an intron-encoded endonuclease, with cleavage sites in *rrl *genes [7,30-33]. Cleavage of *S. paratyphi *C RKS4594 genomic DNA with I-CeuI generated seven fragments (Figure 1). This number is consistent with the previously analyzed *Salmonella *genomes [8], as all *Salmonella *lineages have seven *rrl *genes.

In Figure 1A, there are bright as well as less bright DNA bands, the latter being labeled as combinations of two or three of the seven bright bands. These less bright DNA bands are partial cleavage products showing the neighboring relationships of the seven I-CeuI cleaved DNA segments. Analyses of both complete and partial I-CeuI cleavages resulted in the cleavage map of *S. paratyphi *C RKS4594 in Figure 1B. In designation of the I-CeuI fragments, the same letters were used to label homologous DNA segments in different *Salmonella *lineages. In *S. typhimurium *LT2, the order of the seven I-CeuI fragments is ABCDEFG clockwise, but in *S. paratyphi *C RKS4594 the order is ABCFDEG due to the translocation of fragment F. Translocations of I-CeuI fragments are usually mediated by homologous recombination between pairs of the *rrn *genes [15,16,34,35], which usually have greater than 99% sequence identity. The translocation of I-CeuI F in *S. paratyphi *C RKS4594 led to three hybrid *rrn *operons. The *rrl *genes are used to exemplify the hybrid *rrn *operons in Figure 1B, shown as *rrlC/B*, *rrlE/C *and *rrlB/E*, but recombination may also occur in *rrs*.

### XbaI and AvrII cleavages and transposon Tn*10 *insertion analysis of the genome

**Figure 2:**
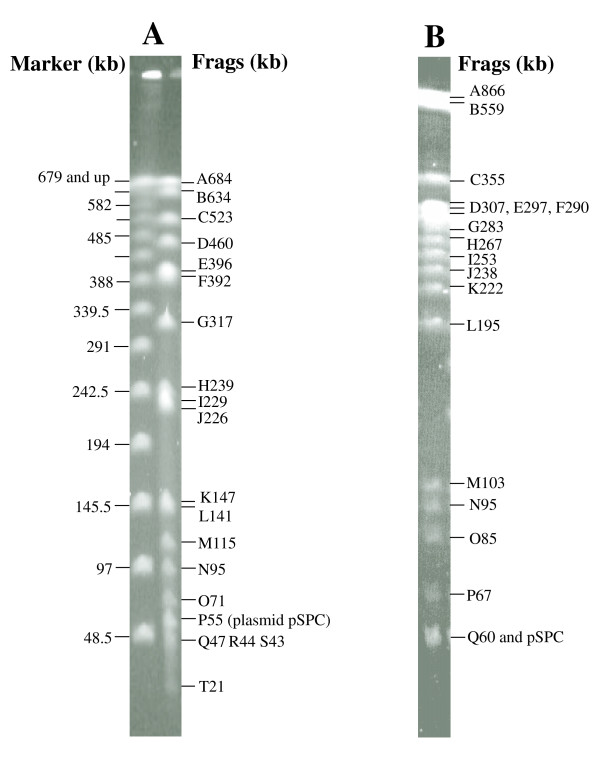
**PFGE separation of cleaved genomic DNA of *S. paratyphi *C RKS4594**. (A), cleavage with XbaI; (B), cleavage with AvrII.

**Figure 3:**
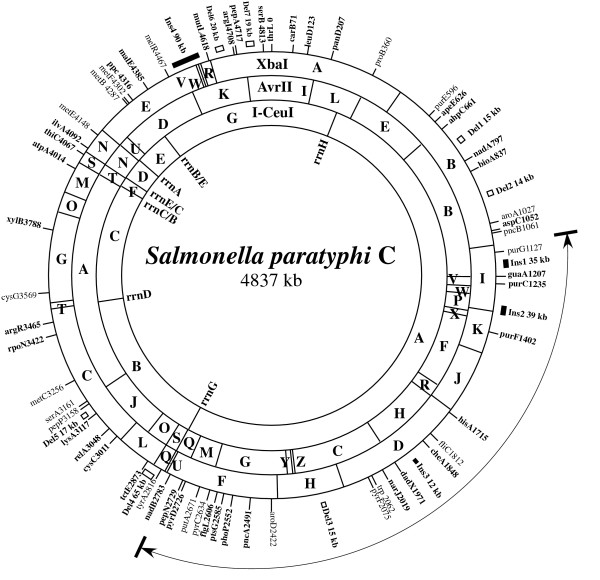
**Genome map of *S. paratyphi *C RKS4594**. The sizes of cleavage fragments on the map are shown to scale, and are listed in Figs 1 and 2. Solid rectangles indicate insertions (Ins1–4) and open rectangles indicate deletions (Del1–7), numbered clockwise, relative to *S. typhimurium *LT2; the sizes are roughly to scale. The gene *thrL *is taken as the beginning of the map for the convenience of comparison with other *Salmonella *maps. The double-headed arrow, with one end being between *pncB *(at 1061 kb) and *purG *(at 1127 kb) and the other between *pepN *(at 2729 kb) and *nadB *(at 2783 kb), covers the region of inversion with respect to the chromosome of *S. typhimurium *LT2.

XbaI cleavage generated 23 fragments, ranging from 684 kb (band A in Figure 2A) to 3 kb (band W, run out of the gel in Figure 2A but seen on other gels). AvrII cleavage generated 26 fragments, ranging from 866 kb (band A in Figure 2B) to 11 kb (band Z, run out of the gel in Figure 2B). To determine the order of fragments in both XbaI and AvrII cleavages and locate genes on the genome of *S. paratyphi *C, we transferred the transposon Tn*10 *that was inserted in known genes in *S. typhimurium *LT2 [36] to the *S. paratyphi *C background by bacteriophage P22 mediated transduction as previously described [19], but with the introduction of O12 antigen-encoding genes to make *S. paratyphi *C sensitive to P22 (details provided in Materials and Methods). Since the Tn*10 *sequence contains both XbaI and AvrII cleavage sites [37], the location of a Tn*10 *insertion can be determined by XbaI or AvrII cleavage and PFGE analyses. DNA from a strain with a Tn*10 *insertion in a specific fragment will lose that fragment, and two fragments will be detected that sum to the size of the lost fragment plus 9.3 kb, which is the size of Tn*10*. We determined locations for 66 genes, as shown in Figure 3.

### A high resolution genome map of *S. paratyphi *C RKS4594

After summarizing experimental results obtained by cleavages with I-CeuI, XbaI and AvrII, Tn*10 *insertion analyses and double digestions (technical details described previously [36]), we constructed a high resolution genome map for *S. paratyphi *C RKS4594 (Figure 3). XbaI fragment P (55 kb) was not mapped on the chromosome; it is a plasmid (pSPC on Figure 2A and 2B) with a copy number of 3 to 5 as judged by brightness of the DNA band when compared with other bands on a PFGE gel. In addition to an XbaI cleavage site, this plasmid also has an AvrII site (See Figure 2B; the plasmid band migrates together with the 60 kb AvrII fragment Q). Most genes are in the same order as in *S. typhimurium *LT2, with the following exceptions. The translocation of I-CeuI fragment F moves the *thiC *gene (and all other genes between *rrnB *and *rrnE*, data not shown here) to a new location; a very large inversion (estimated as 1602 kb) includes *purG *(at 1127 kb on the map) and *pepN *(at 2729 kb on the map). On the genome of *S. typhimurium *LT2, the region clockwise to *pncB *(which, at 1061 kb in *S. paratyphi *C, is just outside the inverted region) is occupied by prophage Gifsy-2 (45 kb in size), and the region counterclockwise to *nadB *(which, at 2783 kb in *S. paratyphi *C, is just outside the inverted region at the other end of the 1602 kb inversion) is occupied by prophage Gifsy-1 (48 kb). The distance between *pncB *and *purG *(66 kb) plus that between *pepN *and *nadB *(54 kb) in *S. paratyphi *C RKS4594 (total 120 kb) roughly equals the distance between *pncB *and *pepN *(47 kb) plus that between *purG *and *nadB *(72 kb) in *S. typhimurium *LT2 (total 119 kb). So, judged by the distances between genes, it seems that two prophages with sizes similar to those of Gifsy-2 and Gifsy-1 are present in *S. paratyphi *C RKS4594, although their identities are unknown. Because the inversion seems to include most genes of the two prophages, and because the prophages are inverted in orientation in *S. typhimurium *LT2, we speculate that the exchange points of the inversion were between genes in the two prophages. At the position of Gifsy-2 in *S. typhimurium *LT2, *S. typhi *CT18 has a different phage sequence, but at the position of Gifsy-1 in *S. typhimurium *LT2, *S. typhi *CT18 does not have a phage sequence [9,10]. A similar inversion has not been observed in other *Salmonella *serovars.

There are several genomic regions with increased distances between genes relative to *S. typhimurium *LT2, which we speculate to be DNA segments acquired from foreign sources during the evolution of *S. paratyphi *C. The first (Ins1) is 35 kb between *purG *and *guaA*, and the second (Ins2) is 39 kb between *purC *and *purF*. It is possible that both are prophages as judged by their sizes, or that one or both might be summations of smaller insertions in those regions from different sources. A 12 kb genomic increase (Ins3) was located in the region between *cheA *and *dadX*. In this region, there is a 50 kb phage-associated DNA segment in *S. typhi *CT18 but not in *S. typhimurium *LT2 [9,10]. It is not known whether the 12 kb is part of the 50 kb phage DNA or whether the whole 50 kb phage DNA is here and there is a 38 kb deletion, collectively giving rise to a net 12 kb increase of DNA in that region.

The largest insertion is the one between *melR *and *mutL*, about 90 kb, in a location similar to that of SPI7 in *S. typhi *[9,14,15]. As it is previously known that the genome of *S. paratyphi *C carries a shorter version of SPI7 [29], we assume that this 90 kb insertion probably is the *S. paratyphi *C version of SPI7.

We also located genomic regions of reduced distances (possible deletions) between genes in *S. paratyphi *C RKS4594 when compared with *S. typhimurium *LT2. Starting from the gene *thr *and moving clockwise, the first possible deletion is one about 15 kb between *ahpC *and *nadA *and the second one is about 14 kb between *bioA *and *aroA*. Whether they contain single deletions or several smaller deletions would both be interesting, especially regarding the gene loss involved, as the absence of some of the genes may be associated with host restriction.

The distance between *aroD *and *pyrF *is 362 kb in *S. typhimurium *LT2 but only 347 kb in *S. paratyphi *C RKS4594. In the same region on the genome of *S. typhi *CT18, there is a 34 kb phage-associated DNA sequence. Depending on whether *S. paratyphi *C RKS4594 contains this 34 kb phage DNA, this organism has a deletion of 15 kb or 49 kb in this region relative to *S. typhimurium *LT2.

*S. typhimurium *LT2 has prophage Fels-2 (35 kb) between genes *tyrA *and *tctE*. In this region, *S. paratyphi *C RKS4594 is 65 kb shorter compared to *S. typhimurium *LT2. If Fels-2 is not present, there should be another 30 kb deletion in *S. paratyphi *C RKS4594. Three additional regions of possible deletions of 17 kb (between *lysA *and *pepP*), 20 kb (between *mutL *and *argI*) and 19 kb (between *pepP *and *serB*) need further characterization. Although we do not completely rule out the possibility that the reduced distances between genes actually resulted from translocation of DNA segments instead of deletions, it is very unlikely, as gene order is highly conserved in *Salmonella *and DNA translocation does not generally occur except those mediated by recombination of *rrn *genes or other repeated elements.

### Plastic genome structure of *S. paratyphi *C

**Figure 4:**
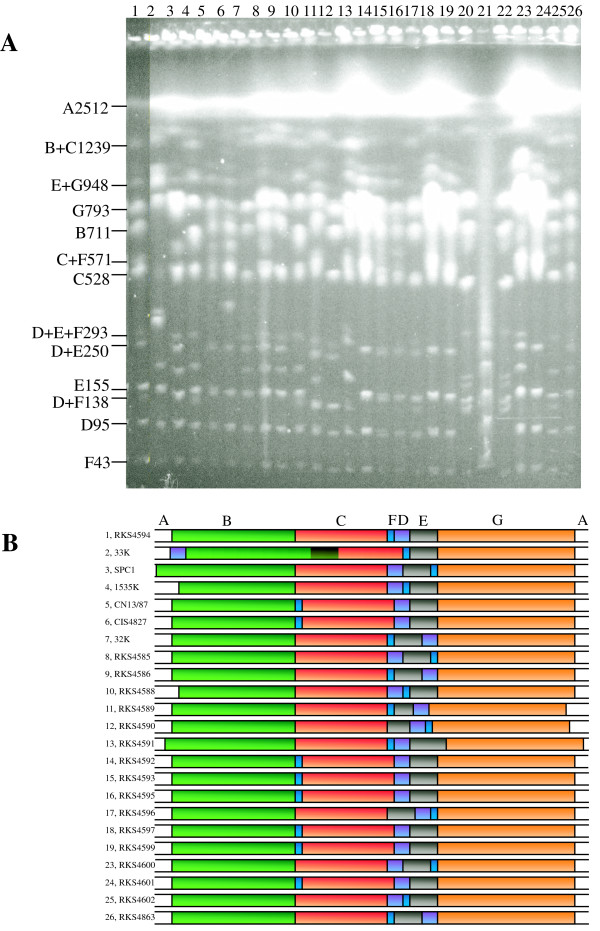
**Diversity in genome structure among wild type strains of *S. paratyphi *C. **(A) PFGE patterns of I-CeuI cleaved genomic DNA from wild type strains of *S. paratyphi *C. Lanes: 1, RKS4594 (IP2/88); 2, RKS4587 (33K); 3, SPC1; 4, 1535K; 5, CN13/87; 6, CIS4827; 7, 32K; 8, RKS4585; 9, RKS4586; 10, RKS4588; 11, RKS4589; 12, RKS4590; 13, RKS4591; 14, RKS4592; 15, RKS4593; 16, RKS4595; 17, RKS4596; 18, RKS4597; 19, RKS4599; 20, *S. typhi *Ty2 (for comparison); 21, λ DNA concatemer as size marker; 22, *S. typhi *Ty2; 23, RKS4600; 24, RKS4601; 25, RKS4602; 26, SA4863. (B) I-CeuI maps of *S. paratyphi *C wild type strains based on data from (A).

To determine whether the genome structure of *S. paratyphi *C is uniform and stable among the populations like that of *S. paratyphi *A [17], or is diverse and plastic like that of *S. typhi *[15,16,35], we made I-CeuI maps on *S. paratyphi *C isolates gathered from different geographical areas over a broad range of time. As shown in Figure 4A, individual *S. paratyphi *C strains have obviously different PFGE patterns, especially regarding the partial cleavage bands, which reflect different orders of the genomic DNA segments (Figure 4B).

Rather strikingly, two strains, RKS4589 and RKS4590, have a 20 kb deletion in a region between *rrlA *and *rrlB*. This region is extremely stable in *Salmonella *and no deletion has been reported in other *Salmonella *lineages. Currently, we do not know what genes have been deleted in these two strains and whether they have identical deletions.

## Discussion

The existence of different typhoid agents causing very similar clinical manifestations presents exceptional opportunities for elucidating their origin(s) and for understanding the molecular mechanisms of host adaptability. Two of the four human typhoid agents, *S. typhi *and *S. paratyphi *A, have been studied at the level of physical genome maps with a focus on genome structure in large numbers of isolates [14-17,35] and at the level of whole genome sequence with a focus on individual genes and their regulation in representative strains [9,11,12]. When compared with *S. typhimurium*, *S. typhi *has over 600 unique genes [9]. It is reasonable to speculate that some of the *S. typhi*-specific genes may be directly involved in typhoid pathogenicity, although cooperation with genes of the "core *Salmonella *genome", *i.e.*, genes shared by most *Salmonella *lineages, should also be important. If one assumes that the typhoid agents share some or many typhoid-associated genes, then comparisons among them should help in identification. Comparative studies between *S. typhi *and *S. paratyphi *A, however, are so far not conclusive, because they each have many genomic features not shared by the other. Therefore, it is necessary to compare more than two *Salmonella *typhoidal lineages to further highlight some possible common genomic features. Additionally, it is also possible that some typhoid agents may have evolved vertically from a common ancestor and others may have acquired typhoid pathogenicity horizontally. Systematic genome comparisons among multiple typhoid agents are rational approaches toward elucidating these evolutionary processes.

The main purpose of this study was to reveal large scale genomic features of a representative *S. paratyphi *C strain and overall genome structure of *S. paratyphi *C populations. On the high resolution physical map of *S. paratyphi *C RKS4594, we resolved four possible genomic insertions, totaling 176 kb, and seven possible deletions, totaling 165 kb, relative to *S. typhimurium *LT2. At this stage, we do not know what genes are contained in the insertions and what genes are involved and deletions, but localization of these insertions and deletions on the genome will significantly facilitate comparative studies among the typhoid agents.

A basic feature of the genome structure of *S. paratyphi *C is its plasticity – large genomic DNA segments are arranged differently in different wild type strains. In strain RKS4594, we first revealed two rearrangement events, including inversion of a 1602 kb segment covering the *ter *region and the translocation of the 43 kb I-CeuI F fragment from its location between I-CeuI E and G as in *S. typhimurium *and most other *Salmonella *genomes to the location between I-CeuI C and D, leading to the order I-CeuI ABCFDEG in *S. paratyphi *C (Figure 3). Diverse genome structures among the *S. paratyphi *C wild type strains representing different populations have resulted from homologous recombination between *rrn *genes in all analyzed strains except RKS4587, in which a very rare inversion occurred between a site in I-CeuI fragment B and a site in I-CeuI fragment C; we are now determining the exact locations and identities of the exchange end points (Liu, on-going project).

The previously analyzed typhoid agents have different situations regarding the stability of genome structure. We first reported the rearranged genome structure of *S. typhi *Ty2 [14,15] and then found that wild type strains of *S. typhi *representing different populations have diverse genome structures; we called this phenomenon "genomic plasticity" [16,34]. Our interpretation of this phenomenon is that large insertions disrupt the physical balance of the genome between *ori *and *ter*, and rearrangements will rebalance the genome [35]. In contrast, the other extensively investigated typhoid agent, *S. paratyphi *A, at both physical map and sequence levels, has a uniform genome structure among different isolates, even though this organism does have insertions totaling 100 kb clustered together on the genome. Interestingly, the 2400 kb inversion, seen in all examined wild type strains of *S. paratyphi *A, nearly completely restored the genomic balance [17], making it unnecessary for the bacteria to further rearrange the genome. The situation with *S. paratyphi *B, on the other hand, seems much more complex, because, unlike *S. typhi, S. paratyphi *A or *S. paratyphi *C, which are all monophyletic [4,14,15,23], bacteria under the name *S. paratyphi *B seem to be genetically diverse. For example, the d-tartrate-positive subline (strains that ferment d-tartrate) causes gastroenteritis instead of typhoid. Kauffmann proposed the name *S. java *for this subline [38], however Le Minor et al. proposed that the name *S. java *be dropped [39]. Even within the d-tartrate-negative and typhoid-causing subline, the diversity in genome structure, as reflected by XbaI and AvrII cleavage patterns, is also significant [20]. Genome sequencing of a representative typhoid-causing strain(s) of *S. paratyphi *B followed by population analysis will provide insights into the phylogenetics and evolution of *S. paratyphi *B as a typhoid agent.

This study demonstrates that *S. paratyphi *C is similar to *S. typhi *in the plasticity of genome structure. However, such a similarity is not indicative of their relatedness. Instead, this similarity suggests a similar route of evolution: acquisition of typhoid causing genes, disruption of physical balance of the genome, and rearrangement of the genome to restore the balance. This route may apply to *S. paratyphi *A as well: the non-plastic feature of its genome may be merely a result of the near complete restoration of the genome balance by the 2400 kb inversion. It is not known at present whether *S. paratyphi *B, monophyletic or polyphyletic as it may turn out to be, has a stable or plastic genome structure; however, it may too have followed a similar route of pathogenic evolution. Revelation of genes that contribute to the typhoid pathogenesis in the individual typhoid agents will update our knowledge on pathogenically similar but phylogenetically not directly related bacteria. For achieving this goal, sequencing of additional *Salmonella *typhoid agents is currently underway in several institutions, and some of the sequencing projects are close to or already at the finishing stages.

This study also revealed an unusual feature of *S. paratyphi *C: unlike *S. typhi *wild type strains that do not have significant DNA content differences except SPI7 that is missing from some *S. typhi *isolates [27], two *S. paratyphi *C strains in this study have 20 kb deletions in a normally extremely stable genomic region between *rrlA *and *rrlB *(Figure 4). This finding suggests that genes in the deleted 20 kb DNA are no longer needed at least in some *S. paratyphi *C strains, or they are not needed by any of the typhoid agents in general. It is possible that, considering the short evolutionary history of the *Salmonella *typhoid agents [40,41], these bacteria may have not yet eliminated all of the non-selected genes, although some stochastic factors, such as integration or excision of prophages that make genomic rebalancing necessary, may have worked to help streamline the genome by removing some of the presumably unneeded genes before they even had a chance to become pseudogenes. These findings may help prioritize genes for functional analyses by excluding large numbers of genes from further study.

## Conclusion

*S. paratyphi *C has diverse genome structures, possibly as a result of large genomic insertions, e.g., SPI7. Although the *Salmonella *typhoid agents may not be more closely related among them than each of them to other *Salmonella *lineages, they may have evolved in similar ways, i.e., acquiring typhoid-associated genes and making genome structure rearrangements. Comparison of multiple *Salmonella *typhoid agents at both single sequenced genome and population levels will facilitate the studies on the evolutionary process of typhoid pathogenesis, especially the identification of typhoid-associated genes.

## Methods

### Bacterial strains

The following 23 *S. paratyphi *C strains were used in this study: RKS4594 (IP2/88), RKS4587 (33K), SPC1, 1535K, CN13/87, CIS4827, 32K, RKS4585, RKS4586, RKS4588, RKS4589, RKS4590, RKS4591, RKS4592, RKS4593, RKS4595, RKS4596, RKS4597, RKS4599, RKS4600, RKS4601, RKS4602, and SA4863; information on these strains can be found at the *Salmonella *Genetic Stock Center. Culture conditions were as described previously [36].

### Isolation, endonuclease cleavage, and electrophoretic separation of bacterial genomic DNA

Bacteria were embedded in agarose to avoid shearing of genomic DNA during isolation, as described previously [21]. Endonuclease cleavage with I-CeuI, XbaI and AvrII and separation of the cleavage fragments by pulsed field gel electrophoresis (PFGE) were also described previously [7,14].

### Transfer of Tn*10 *insertions through bacteriophage P22 mediated transduction

A large number of Tn*10 *insertions into genes with known functions have been established in *S. typhimurium *LT2 in a great range of laboratories. We transferred Tn*10 *insertions from *S. typhimurium *LT2 to *S. paratyphi *C by bacteriophage P22 mediated transduction to locate these genes through homologous recombination. As P22 infects only *Salmonella *serovars that express O12 antigen but *S. paratyphi *C does not have O12, we transferred the cosmid pPR1347 [42] carrying the genes coding for the long chain O antigen into *S. paratyphi *C RKS4594 to make it sensitive to P22. The P22 transduction experiments have been described previously [19]. Briefly, we made P22 lysates from a selected set of Tn*10 *insertion mutants of *S. typhimurium *LT2 by growing a 3 ml overnight culture in LB broth of these selected Tn*10 *mutants and inoculating these cultures with phage P22 at a multiplicity of infection of 1:100, followed by co-incubation for 6 hours. After removal of the cell debris by centrifugation, the lysates, which contained approximately 10^11^pfu/ml of phage particles, were ready for use in the transduction. For transferring the Tn*10 *insertions to *S. paratyphi *C, we spread 100 μl overnight culture of *S. paratyphi *C carrying pPR1347 and 10 μl lysate onto an LB plate containing tetracycline. A colony was picked up and re-streaked on another tetracycline plate for single colony isolation. One colony from the second tetracycline plate was used for phenotype tests and mapping.

### Enzymes and chemicals

I-CeuI, AvrII and SpeI were purchased from New England BioLabs; XbaI and proteinase K were from Roche. Most other chemicals were from the Sigma Chemical Co.

## Authors' contributions

GRL and DQ initiated the PFGE experiments; WQL, GRL and JQL completed the physical mapping; XYH, JD, GMX, LW and FMZ were involved in construction of a P22 sensitive strain of RKS4594 by introducing into the bacteria the cosmid pPR1347 that carries O12 encoding genes, bacterial culture, Tn*10 *insertion inactivation, DNA isolation, and phenotype testing; RNJ and SLL coordinated the work; WQL and SLL produced the manuscript.
